# Association of Television Viewing Time With Overweight/Obesity Independent of Meeting Physical Activity Guidelines: Do Joint Exposures Yield Independence?

**DOI:** 10.2188/jea.JE20130073

**Published:** 2013-09-05

**Authors:** Meagan E. Stabler, Peter R. Giacobbi, Desta B. Fekedulegn

**Affiliations:** 1 Department of Epidemiology, School of Public Health, West Virginia University, Morgantown, West Virginia, USA; 2 Department of Sport Sciences with Joint Appointment to Department of Epidemiology, School of Public Health, West Virginia University, Morgantown, West Virginia, USA; 3 Biostatistics and Epidemiology Branch, Health Effects Laboratory Division, National Institute for Occupational Safety and Health, Centers for Disease Control and Prevention, Morgantown, West Virginia, USA

Dear Dr. Tomotaka Sobue (editor):

Inoue and colleagues^1^ published results from a cross-sectional mail survey that examined the joint associations of television viewing time and a categorical estimate of moderate to vigorous physical activity (MVPA) with overweight/obesity in older Japanese adults.^1^ While it is fitting to draw conclusions regarding the joint associations of television viewing time and MVPA with body composition, the authors inappropriately concluded that spending less time watching TV reduced the “risk” of being overweight or obese “independent” of meeting physical activity guidelines (MVPA). The purpose of this editorial is to question the use of this terminology in their conclusion from a statistical and epidemiological perspective. We propose that the effect of TV viewing time on obesity, independent of MVPA, could not be inferred from the statistical model they fit and we therefore suggest an alternative model specification that would test the independent effects.

The authors created a new exposure variable that combined the levels of television viewing time (low, high) and MVPA (insufficient, sufficient) and classified the study participants into 4 categories. They fit logistic regression models relating overweight/obesity (≥25 kg/m^2^) to the 4-level exposure variable, adjusting for potential confounders. Odds ratios comparing the odds of being overweight/obese were computed for each combination of TV viewing time and MVPA using high TV viewing time and insufficient MVPA as the referent group. However, it is generally inappropriate to conclude independent contributions of television viewing and MVPA to body composition, unless both exposure variables were being controlled for in the same model as separate variables.

As demonstrated by Merikanto et al (2012), to properly examine the association of TV viewing time and obesity independent of MVPA, the appropriate statistical modeling approach includes fitting a model relating obesity to TV viewing time, MVPA, and the interaction between the 2 predictors.^2^ If the interaction term is significant, then a model that examines the effect of TV viewing time at each level of MVPA could be fit. In the absence of an interaction effect, a main-effects model that contains the 2 predictors in the same model would enable one to make appropriate inferences regarding independent effects of the factors. Further discussion on interaction between two exposures is presented by Mutsert et al (2009).^3^

The study by Merikanto et al (2012) demonstrates appropriate use of a statistical modeling strategy for testing the independent effects of 3 exposure variables (chronotype, sleep duration, and sufficient sleep) on cardiovascular disease-related morbidity and type 2 diabetes. In their analysis, Merikanto et al (2012) fit a multivariate logistic regression model that contained all 3 predictor variables simultaneously.

In conclusion, the authors accomplished their study aim of testing the joint association of television viewing and MVPA with body composition. However, we believe they inaccurately used the term ‘independent’ to interpret their results when they concluded that less TV viewing time is associated with reduced risk of obesity “independent” of meeting PA guidelines. Potential solutions are to utilize prevalent-sensitive measures (eg, avoid risk-like wording) and avoid conclusions that claim independence between variables transformed as joint-exposures.

## References

[1] Inoue S, Sugiyama T, Takamiya T, Oka K, Owen N, Shimomitsu T. Television viewing time is associated with overweight/obesity among older adults, independent of meeting physical activity and health guidelines. J Epidemiol. 2012;22:50–6. 10.2188/jea.JE20110054 22156288 PMC3798580

[2] Merikanto I, Lahti T, Puolijoki H, Vanhala M, Peltonen M, Laatikainen T, . Associations of chronotype and sleep with cardiovascular diseases and type 2 diabetes. Chronobiol Int. 2013;30(4):470–7. 10.3109/07420528.2012.741171 23281716

[3] De Mutsert R, Jager KJ, Zoccali C, Dekker FW. The effect of joint exposures: examining the presence of interaction. Kidney Int. 2009;75(7):677–81. 10.1038/ki.2008.645 19190674

